# Carbon Dots–Biomembrane Interactions and Their Implications for Cellular Drug Delivery

**DOI:** 10.3390/ph16060833

**Published:** 2023-06-02

**Authors:** Barbara Mavroidi, Archontia Kaminari, Elias Sakellis, Zili Sideratou, Dimitris Tsiourvas

**Affiliations:** 1 Institute of Biosciences and Applications, National Centre for Scientific Research “Demokritos”, 15310 Aghia Paraskevi, Greece; bmavroidi@bio.demokritos.gr; 2 Institute of Nanoscience and Nanotechnology, National Centre for Scientific Research “Demokritos”, 15310 Aghia Paraskevi, Greece; a.kaminari@inn.demokritos.gr (A.K.); e.sakellis@inn.demokritos.gr (E.S.); z.sideratou@inn.demokritos.gr (Z.S.)

**Keywords:** carbon dots, biomembranes, lipid bilayers, membrane permeability, doxorubicin

## Abstract

The effect of carbon dots (CDs) on a model blayer membrane was studied as a means of comprehending their ability to affect cell membranes. Initially, the interaction of N-doped carbon dots with a biophysical liposomal cell membrane model was investigated by dynamic light scattering, z-potential, temperature-modulated differential scanning calorimetry, and membrane permeability. CDs with a slightly positive charge interacted with the surface of the negative-charged liposomes and evidence indicated that the association of CDs with the membrane affects the structural and thermodynamic properties of the bilayer; most importantly, it enhances the bilayer’s permeability against doxorubicin, a well-known anticancer drug. The results, like those of similar studies that surveyed the interaction of proteins with lipid membranes, suggest that carbon dots are partially embedded in the bilayer. In vitro experiments employing breast cancer cell lines and human healthy dermal cells corroborated the findings, as it was shown that the presence of CDs in the culture medium selectively enhanced cell internalization of doxorubicin and, subsequently, increased its cytotoxicity, acting as a drug sensitizer.

## 1. Introduction

Nanoparticles of a wide variety in terms of chemical composition, size, shape, surface functional groups, and surface charge have been extensively developed and studied in life sciences, predominantly for drug delivery, including cell or tissue targeting, bioimaging, and diagnosis [1]. Particular attention has been given to those nanoparticles that have minimal cell toxicity, hemocompatibility, biodegradability, and clearance. To better understand and, ultimately, to predict the behavior of nanoparticles in real biological systems, knowledge about their interaction with cell membranes is essential [2]. To this end, investigation of their interaction(s) with cell membrane models is suggested as a first useful step, as such interaction(s) can be studied and quantified by employing well-characterized systems and a precisely controlled experimental environment [3].

Among the nanoparticles scrutinized for their potential in medical applications, carbon dots (CDs), a group of fluorescent and photostable nanocarbon-based materials with sizes ranging between 2 and 10 nm, are attracting attention, as they are typically water-soluble and chemically inert, with minimal toxicity and biocompatibility [4,5], while their preparation is, in most cases, environmentally friendly. These properties render CDs ideal for biomedical applications, including drug and gene delivery [6,7], biological imaging [8,9,10,11,12], and, ultimately, applications as theranostic agents [13]. While a large variety of carbon dots differing in starting materials, method of production, and functionalization have been developed and described in the literature—some of which are no longer non-toxic or biocompatible—we focused on nitrogen-doped (N-doped) carbon dots that are among the most widely studied CDs because they utilize low cost starting materials, including organic acids (such as citric acid) and nitrogen compounds (usually ammonia, ethylenediamine, or urea), are easily produced, have high quantum yields, and, more importantly, their minimal toxicity and hemocompatibility is well-established [5,6,7,14,15,16,17,18].

Despite the evidence provided of CDs’ low cell toxicity, as established by a variety of relevant methods, including cell viability assays, mitochondrial superoxide or mitochondrial membrane measurements, ROS generation, or annexin–propidium iodine assays, there is still a general concern about the effects these nanometer-sized particles might have on human health. To this end, it is initially essential to acquire more data on the effect they have on the membranes of cells, as this is the first stage of their interaction. Indeed, this issue has been the subject of recent studies related to a variety of nanoparticles [19,20,21,22], which highlighted the usefulness of cell membrane models, as they facilitate the control of experimental parameters, the ease of reliable data acquisition, and the possibility of providing information on the structural and thermodynamic properties of the interacting system [3,23,24,25]. Liposomes, which are spherical vesicles consisting of a phospholipid bilayer membrane, are the most commonly used models that mimic biological membranes. Their usefulness has been shown in studies that proved, for example, that pore formation is induced in phosphatidylcholine membranes by the presence of spherical silica nanoparticles [26] or that the nonspecific adsorption of charged polymeric nanoparticles onto phosphocholine bilayers can lead either to local gelation, when they are negatively charged, or to local fluidization when they are positively charged [27]. Similar experimental studies on carbon dots’ interaction with phospholipid membranes are few and have been mainly related to hydrophobic (non-water soluble) carbon dots and their effects on bilayer fluidity and lipid dynamics [28,29,30,31], while a molecular-dynamics simulation study employing water-soluble hydroxyl-functionalized carbon dots showed that CDs have no impact on the bilayer structure, although it was shown that water permeation increases when the carbon dot is allowed to penetrate into the hydrophobic tail region of the lipid bilayer [32].

In this study, we evaluated the effect of nitrogen-doped (N-doped) carbon dots on the structural properties and thermodynamic properties of phospholipid liposomal bilayer membranes. Mixed liposomes consisting of the biomembrane-relevant lipids dipalmitoyl phosphatidylcholine (DPPC) and the negatively charged dipalmitoyl phosphatidylglycerol (DPPG) at a low molar content (5%) were employed, mimicking the negatively charged surface of cells, in order to simulate the interaction of the slightly positive CDs with a cell’s surface. DPPC and DPPG lipid monolayers have been used as membrane models to probe the interactions of NPs, oligonucleotides or plasma proteins with lipid membranes [3,33,34,35]. An added advantage of using DPPG is that DPPC–DPPG-mixed bilayers, at neural pH, exhibit almost ideal miscibility in the gel and in the liquid crystalline state [36,37,38]. In addition, a negatively charged PEGylated phospholipid, also at a low molar content (5%), was added to the bilayer to stabilize the membrane, due to the presence of the protective polyethylene glycol layer, and to simulate cell membranes, which are known to be decorated with glycosaminoglycans (GAGs), which are the negatively charged, long polysaccharides linked to glycosylated membrane proteins that overcoat all cells [39]. The binding mode of the lipid–CDs interaction was studied by monitoring the effect of the CDs on the z-potential, size, and the gel-to-liquid crystalline lipid phase transition of the liposomes. In addition, we prepared doxorubicin (DOX)-encapsulating liposomes and analyzed bilayer permeability by monitoring DOX release from the liposomes, mediated by increasing CD concentrations. Finally, we examined whether the observed membrane permeability enhancement can also be observed in vitro, employing both cancerous and non-cancerous cell lines.

## 2. Results

The preparation by microwave irradiation of citric acid and ethylenediamine and the characterization of nitrogen-doped CDs is described in detail in our previous publication [18]. Despite that fact that these CDs are nitrogen-doped, having 1.20 ± 0.02 mmol NH_2_/g of primary amino groups [18], they are also characterized by the presence of both carboxylic and hydroxyl groups on their surface due to the selection of the starting compounds. This results in a slightly positive surface charge with a z-potential of 2.5 ± 1.2 mV at isotonic pH 7.4 PBS buffer.

The effect of nitrogen-doped CD_S_ on model bilayer membranes was investigated by preparing slightly negative charged liposomes that mimicked the surface of cells, by incorporating a low amount (5 mol%) of the negatively charged phospholipid DPPG in PEGylated DPPC:DSPE-PEG liposomes. It was anticipated that their interaction would be primarily of electrostatic origin, as there are no complementary sites for specific interactions between the two interacting oppositely charged surfaces. At first, the adsorption of CDs on the negatively charged bilayer membrane was examined after their interaction at different concentrations, temperatures, and media, followed by monitoring the changes in the structural and thermodynamic properties of liposomes in the presence of CDs at various concentrations.

### 2.1. Adsorption of CDs on Liposomes

**Figure 1:**
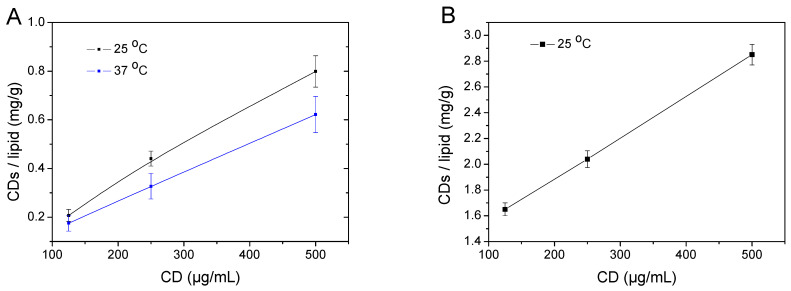
Carbon dots (CDs) adsorbed on DPPC:DPPG:DSPE-PEG liposomes in PBS (**A**) or water (**B**) as a function of their concentration in the outer medium. The DPPC:DPPG:DSPE-PEG liposomes after incubation with CDs in PBS were isolated by ultracentrifugation and the content of CDs was analyzed by fluorescence spectroscopy. Data were obtained from three different independent sets of experiments (the lines are only drawn as guides).

We observed that upon addition of a CDs solution in PBS, at increasing concentrations (125, 250, and 500 μg/mL), to the liposomal dispersions in PBS, the primarily electrostatic interaction between the positively charged CDs with the negatively charged liposomal surfaces resulted in increasing quantities of CDs that were associated on their surface. Indeed, after removal of the free (non-attached) CDs from the medium employing ultracentrifugation, washing with PBS to ensure complete removal of non-attached CDs, and subsequent determination of CDs in the resulting suspension, it was found that the CDs concentration on liposomes scaled almost linearly with the initially added CD concentrations (Figure 1). Repeating these experiments at 37 °C, the same trend was observed, although the determined quantity of CDs attached on liposomes was considerably reduced (ca. 80% reduction). It should be noted that when the same set of experiments was performed in water, the quantity of CDs adsorbed on liposomes was, in all cases, significantly higher (~4-fold increase). Apparently, the interaction is more effective in pure water than in PBS, as it is known that high ionic strength results in screening ionic interactions and in the removal of non-specifically bound entities. The effect of both temperature and the ionic strength of the medium suggested that electrostatic interactions are primarily at play, although some specific interactions could not be ruled out, as even in very unfavorable conditions (high ionic strength and temperature), a number of CDs were still attached.

### 2.2. Effect of CDs on the Structural Properties of Liposomes

**Figure 2:**
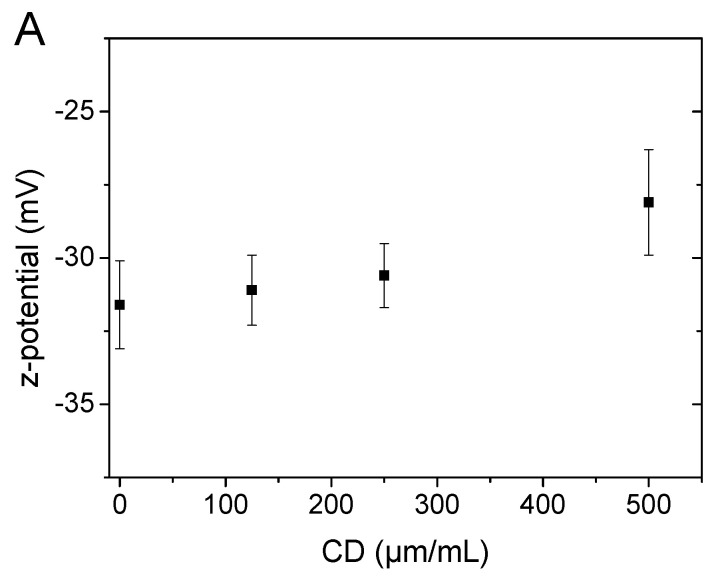

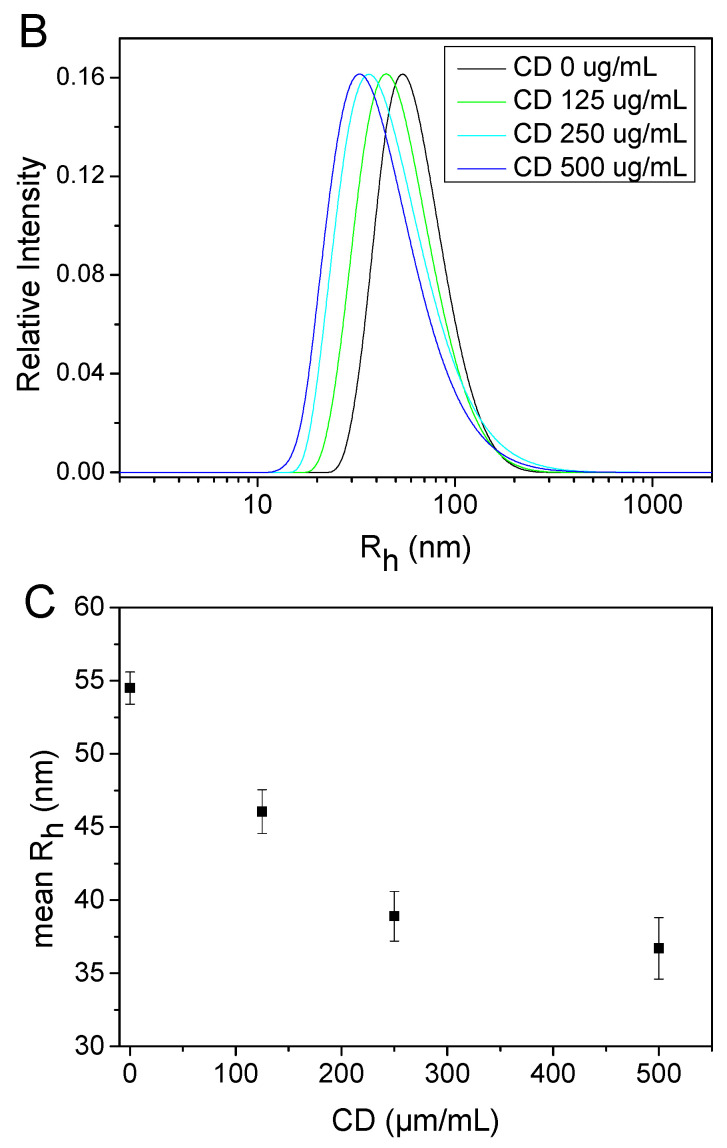
(**A**) Z-potential of DPPC:DPPG:DSPE-PEG liposomes in PBS as a function of CDs’ concentration in the outer medium. (**B**,**C**) Size variation of DPPC:DPPG:DSPE-PEG liposomes in PBS as a function of CDs’ concentration in the outer medium: (**B**) hydrodynamic radius distributions of unilamellar vesicles obtained by CONTIN analysis of DLS measurements and (**C**) apparent mean hydrodynamic radii vs. CDs’ concentration. All data were obtained from three different independent sets of experiments.

The presence of CDs at the outer medium of DPPC:DPPG:DSPE-PEG liposomes in PBS had a clear and substantial effect on their z-potential and size distribution, compared to the original liposomes. The surface charge of liposomes, as expected, increased with the increasing of the concentration of CDs in the medium: the z-potential values of initial liposomes (−31.6 ± 1.9 mV) upon addition of CDs gradually increased to −30.6 ± 1.1 mV at 250 μg/mL and to −28.1 ± 2.1 mV at 500 μg/mL (Figure 2A). The increase in z-potential values was definitely moderate, given the fact that CDs have only slightly positive values (2.5 ± 1.2 mV). On the other hand, the registered mean hydrodynamic sizes from three different independent experiments (Figure 2B,C) revealed a distinct and significant size reduction with increasing CDs concentrations, at least up to 250 μg/mL. Indeed, the mean hydrodynamic radii of the liposomes, from their original value of 54.5 ± 1.2 nm, decreased in size to 38.9 ± 1.7 nm at 250 μg/mL of CDs, with a further slight decrease to 36.7 ± 2.1 nm at 500 μg/mL.

The obtained results can be explained either in terms of an enlarged head group size of the outer monolayer, due to the incorporation of CDs, which would result in the observed change in liposomal membrane curvature, or to the increase in the osmolarity of the solution, due to the addition of CDs in the outer aqueous phase. The latter option would result in osmotic deflation due to the low permeability of the membrane [40]. However, it should be noted that the effect on the osmolarity of the PBS solution (~300 mOsm/L, equivalent to 0.9% *w*/*v* saline solution) of the nanoparticles in our study with a maximum concentration of 0.05% *w*/*v* (in the case when 500 μg/mL of CDs are employed) was minimal and, therefore, this explanation was not credible. On the other hand, it was shown both experimentally and theoretically [41,42,43] that the size of counterions determine the size of the vesicles: indeed, changes in counterion size affects head group curvature, leading to increased head group size and inter-vesicle repulsion, and decreases the size of the liposomes. Thus, it is reasonable to assume that CDs acting as counterions of the negatively charged phospholipid entities exhibit a similar effect by being positioned near or even partly inside the outer part of the bilayer.

In another approach, it has been suggested that during liposome preparation in buffers (the phenomenon is not observed when liposome preparation takes place in pure water) the shearing forces induced by the applied pressure during the passage of vesicles through the pores of the membrane created cylindrical bilayer forms, leading to non-spherical (oval or sausage-shaped) vesicles [44,45]. The liposomal membrane tends to assume a spherical shape, which is the thermodynamically lowest-energy state, but this is not possible as it requires an increase in the inner volume, which is countered by the osmotic force of common buffers [45]. Since the analysis of the autocorrelation function obtained by DLS assumes that the nanoparticles are of a spherical shape, in the case of non-spherical liposomal dispersions, the method is expected to overestimate the hydrodynamic size [46]. The liposomal membrane tends to assume a spherical shape and it is safe to assume, as above, that the positioning of the nanoparticles in the bilayer would modify the membrane curvature and relieve the strain, or even render the membrane more permeable (see also Section 2.4). This would lead to the influx of water in the liposomal interior and, finally, to the rounding up of the liposomes that are manifested as lower DLS values. To verify this hypothesis, the same set of experiments was conducted in pure water under otherwise identical conditions. It was found that the liposomal radii were in all cases 34.0 ± 2.0 nm, with no significant decrease in their size in the presence of CDs, which was within experimental error. Only in the case of the highest CD concentration of 500 μg/mL were the registered radii found to be slightly smaller (32.5 ± 1.5 nm), which indicated some effect on the size that was, however, statistically non-significant.

### 2.3. Effect of CDs on the Thermodynamic Properties of Liposomal Bilayer

**Figure 3:**
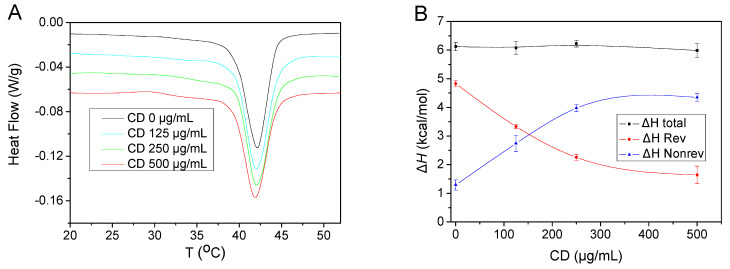

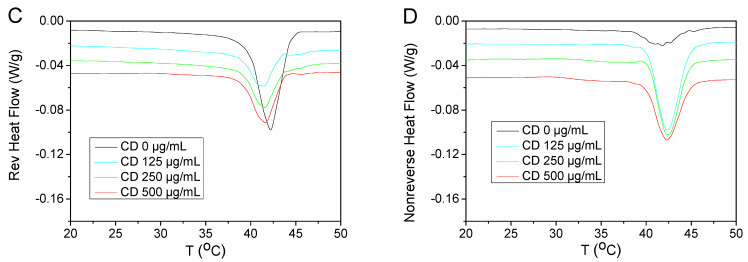
Temperature-modulated DSC analysis of the main phase transition of liposomal DPPC:DPPG:DSPE-PEG formulations in the presence of various CD concentrations in the outer PBS medium: (**A**) Total heat flow profiles of liposomal dispersions during the first heating cycle (endo down); (**B**) Total, reversing and non-reversing enthalpies of the main phase transition. Data were obtained from three different independent sets of experiments. (**C**) Reversing heat flow signals of liposomal dispersions during the first heating cycle (endo down). (**D**) Non-reversing heat flow signals of liposomal dispersions during the first heating cycle (endo down). The heating scan rate for all of the thermographs was 2 °C/min.

DSC analysis was employed to obtain information of the physical state of bilayers in the presence of CDs in the outer aqueous phase. The results showed that all samples examined underwent the main gel-to-liquid crystalline transition, *T*_m_, at 42.1 ± 0.2 °C, irrespective of the presence or absence of the CDs in the outer phase (Figure 3A). In addition, the width of the main transition remained constant in all cases. The registered temperature was in accord with the *T*_m_ values for DPPC:DSPE-PEG liposomes reported in the literature [47,48,49], as it was reasonable to assume that the presence at 5 mol% of DPPG, which has also a similar *T*_m_ to DPPC (~42 °C) [50,51], was not expected to modify the *T*_m_. There was a slight reduction of *T*_m_ in the case of the higher CDs’ concentration which was within experimental accuracy. In line with the above, the corresponding heat capacity profiles (Figure S3) showed that the heat capacity maximum was unaffected; only at high CD concentrations (250 and 500 μg/mL) did it shift slightly (by about 0.6 degrees) to lower temperatures, although it should be emphasized that the observed variations were small and close to the experimental error.

The enthalpies, Δ*H*, of the main lipid phase transition were, in all cases, 6.1 ± 0.1 kcal/mol. This value was within experimental error, unaffected by the presence of CDs, although again there was a slightly reduced registered value when the higher CD concentration was employed, as shown in Figure 3B. The corresponding literature values for 100 nm DPPC liposomes were reported to be 7.5 ± 0.5 kcal/mol [47]. Taking into consideration that the presence of DSPE-PEG is known to reduce the enthalpy of the main transition, and that the presence of DPPG is also expected to further reduce this value [52,53,54], the obtained Δ*H* values were quite reasonable and suggested a barely detectable effect of CDs on the physical state of bilayers. However, it is known that the main thermal transition can be complex, and further insight of the processes taking place during this transition can be gained through the use of temperature-modulated differential-scanning calorimetry [55]. Both the reversing and non-reversing heat flows of the respective thermograms are shown in Figure 3C,D, respectively, while the corresponding enthalpies of the endothermic signals are shown in Figure 3B. It is evident that there is a key difference in the melting process between the pure liposomal dispersions and the liposomal dispersions with CDs present in their outer aqueous phase. It should be noted that this difference was repeatedly observed in all repetitions of this series of experiments, as well as during both the first and second heating runs. The melting of pure liposomal dispersions was mainly reversing in nature (the reversing enthalpy is ~80% of total enthalpy), while in the presence of CDs there was a gradual and clearly notable increase in the non-reversing character of this transition. It should be noted that this increase of the non-reversing character of the main lipid phase transition was observed repeatedly in three different sets of experiments.

Although, to our knowledge, there has been little analysis of MDSC traces for liposomes, it is well established that the melting process in general is typically observed in the reversing heat signal [56,57]. Indeed, the enthalpy of melting of the transition from the main gel (crystalline-like) phase to the liquid crystalline phase (P_β_ → L_α_) was expected to be found mainly in the reversing signal, given that the gel-to-liquid crystalline transition in the case of phosphatidylcholines is the result of the cooperative melting of the hydrocarbon chains of the lipid molecules and is fully reversible and undergoes a rapid process that is not kinetically limited [58]. In line with this is the fact that MDSC analysis of the melting of low-molar-mass liquid crystals or of polymeric liquid crystals indicated a fully reversible transition that was evident in the reversing signal of MDSC [57]. On the other hand, kinetic processes that take place during the transition will be detectable in the non-reversing signal [56], as was observed whenever CDs were present in the aqueous medium. Especially in the case of high CD concentration, the thermal transition is primarily kinetically controlled, appearing in the non-reversing signal (Figure 3B,D). This indicates that the presence of CDs in the bilayer affects the kinetics of the melting process. In keeping with the analysis performed in the cases when non-reversing melting of polymers was observed [57], we tentatively attribute this kinetic effect to chain melting in the (at least partial) localization of CDs within the hydrocarbon chain sublayer of the phospholipids, so that upon temperature increase to the melting temperature, they affect the molecular mobility of the aliphatic chains.

### 2.4. Effect of CDs on the Permeability of Liposomal Bilayer

**Figure 4:**
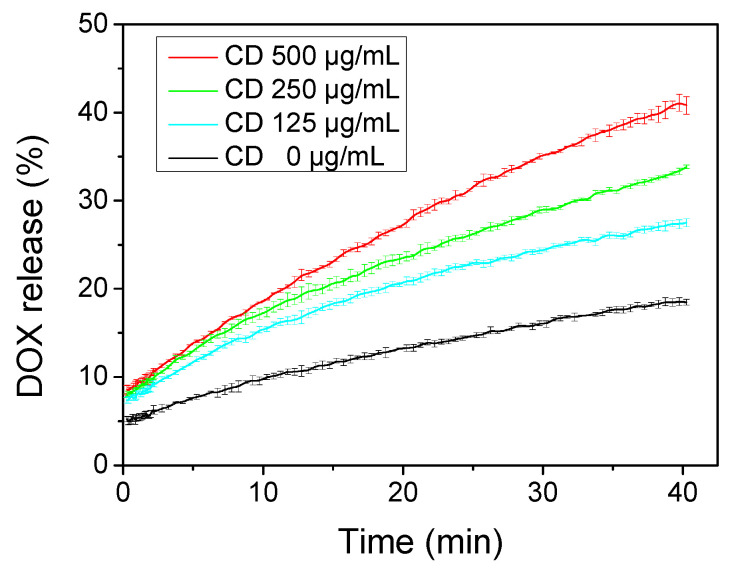
Time-dependent DOX release at 37 °C from DPPC:DPPG:DSPE-PEG unilamellar liposomes in the presence of various CD concentrations in the outer PBS medium. Measurements were taken every 0.1 min during the two minutes of incubation and every 0.5 min afterwards. Data were obtained from three different independent sets of experiments.

The effect of CDs on the permeability of DPPC:DPPG:DSPE-PEG bilayers was assessed by actively loading DOX in liposomes and monitoring its release in the outer phase in the presence of CDs at various concentrations. DOX, a well-known and widely used anticancer agent, was selected both because of its intrinsic fluorescence at low concentrations (i.e., after its release in the external aqueous phase) and its self-quenching properties at high concentrations (i.e., at concentrations attained in the liposomal interior), as well as the ease of translating these experiments in in vitro cell studies, where we could also probe its cell transport indirectly by quantifying cell internalization. The release profiles of encapsulated DOX at 37 °C, either in the absence or in the presence of increasing concentrations of CDs (up to 500 μg/mL), were continuously monitored at 37 °C for a period of 40 min (Figure 4). The registered profiles followed a similar pattern, although it was clear that the original DPPC:DPPG:DSPE-PEG liposomes in the absence of CDs had the lowest release rate. In this case, DOX release reached a maximum value of ca. 18%, while in the presence of CDs even from the beginning of each experiment, i.e., from the first 10 s after the addition of liposomes, DOX release was substantially increased, reaching values of up to 40% withing 40 min. The observed increase in membrane permeability with increasing CD concentration was reflected in the apparent release rate constants. By assuming a (pseudo) first-order release rate that is typically employed to analyze drug release from liposomal formulations (see Experimental Section 4.5.2), the corresponding rate constants, k, were obtained (Table 1) by fitting the release data during the whole-time frame available. It was evident that an increase in CD concentration resulted in an increase in the corresponding rate constants, and that this increase was more pronounced at 500 μg/mL. Release data could be also be examined to determine whether they follow the “square root of time” release kinetics, as suggested and theoretically treated by Higuchi [59]. As shown in Figure S4A a linear relationship of release data was observed after the first 9–10 min, suggesting that after this initial time period the systems reached a so-called pseudo-steady-state, where the cumulative amount of drug released is directly proportional to the square root of time. Therefore, it is also possible to analyze the last part of the release curves employing the Korsmeyer–Peppas equation [59]. The diffusional release exponent n (Table 1) of this equation, as derived for each release curve (Figure S4B), was in most cases close to the theoretical value of 0.43 for carriers of sphere geometry, which indicated that the release mechanism controlling this time period was Fickian diffusion; the considerably higher value obtained (0.59) when CD concentration was 500 μg/mL was characteristic of “anomalous transport”, most likely suggesting that both diffusion-controlled and relaxation-controlled release mechanisms are characterizing this particular case [59].

### 2.5. The effect of CDs on Doxorubicin Cell Internalization and Toxicity

**Figure 5:**
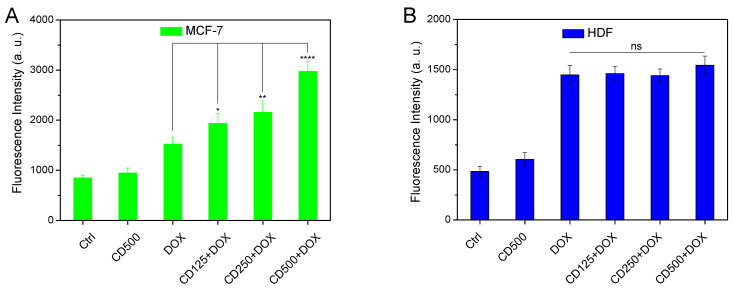
Doxorubicin internalization in MCF-7 cells (**A**) and human dermal fibroblasts (HDF, (**B**). Cells in 96-well plates were incubated at 37 °C and treated with CDs (500 μg/mL), DOX (3 μΜ), and increasing concentration of CDs (125, 250, and 500 μg/mL) for 1 h before adding DOX (3 μM). After 3 h, the wells were washed with RPMI without phenol red and DOX concentration was measured with an Infinite M200 plate reader (λ_ex_ = 510 nm, λ_em_ = 580 nm) and expressed as fluorescence intensity in arbitrary units (a.u.). The results are shown as the mean ± SD for at least three independent experiments and were analyzed using a Student’s *t*-test (* *p* < 0.05, ** *p* < 0.01, **** *p* < 0.0001, ns not significant). Statistical analysis is not shown if it was not considered significant (*n* = 3).

To examine whether the increase in the permeability of phospholipid bilayers in liposomes could also be observed in cell membranes, we proceeded with in vitro cell internalization experiments of DOX in the presence of various concentrations of CDs. Either MCF-7 cancer cells or non-cancerous human dermal fibroblasts (HDF) were treated with various concentrations of CDs (125, 250, and 500 μg/mL) for 1 h before adding DOX (3 μM) for 3 h. In addition to the control, the cells were also treated with DOX (3 μM) and CDs (500 μg/mL) for the same time periods, for comparison purposes. After washing the cells, DOX uptake was quantified by registering the DOX-dependent fluorescence emission (Figure 5).

Compared with the free-DOX treatment, the pre-treatment of MCF-7 cells with CDs resulted in a DOX uptake increase in a dose-dependent manner; even the lowest concentration of CDs (125 μg/mL) led to a 25% increase in DOX uptake, while when the highest CDs concentration was employed, DOX internalization was almost doubled (95% increase, Figure 5A). Interestingly, the same treatment of human dermal fibroblasts (HDF) did not follow the same pattern (Figure 5B). In that case, DOX internalization was not affected at all by the presence of CDs, but remained, within experimental accuracy, constant and lower than that observed when free DOX was administered in cancerous cells. We tentatively attributed this difference to the difference in the cell membrane potential of cells, as it is well established that cancer cells are characterized by a more negative surface charge than non-cancerous cells [60,61,62]. Therefore, if carbon dots interact electrostatically with cell surfaces, as in the case of liposomes, this is expected to selectively increase the cell membrane permeability of cancerous cells, compared to non-cancerous cell lines.

**Figure 6:**
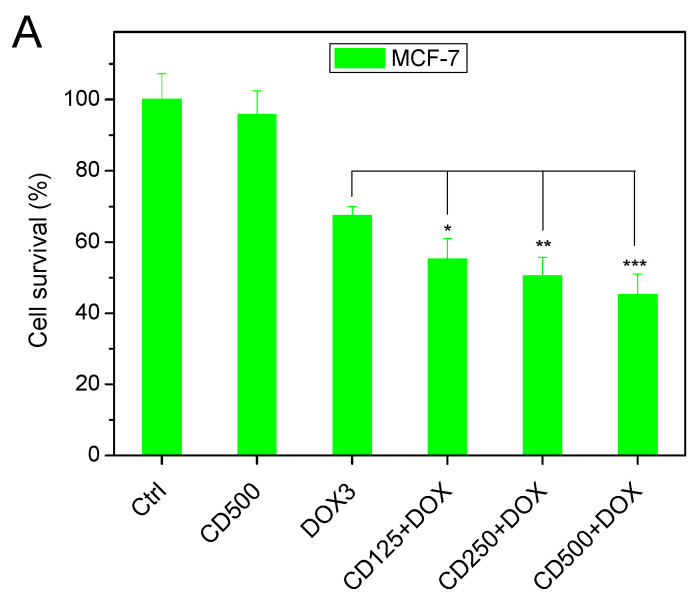

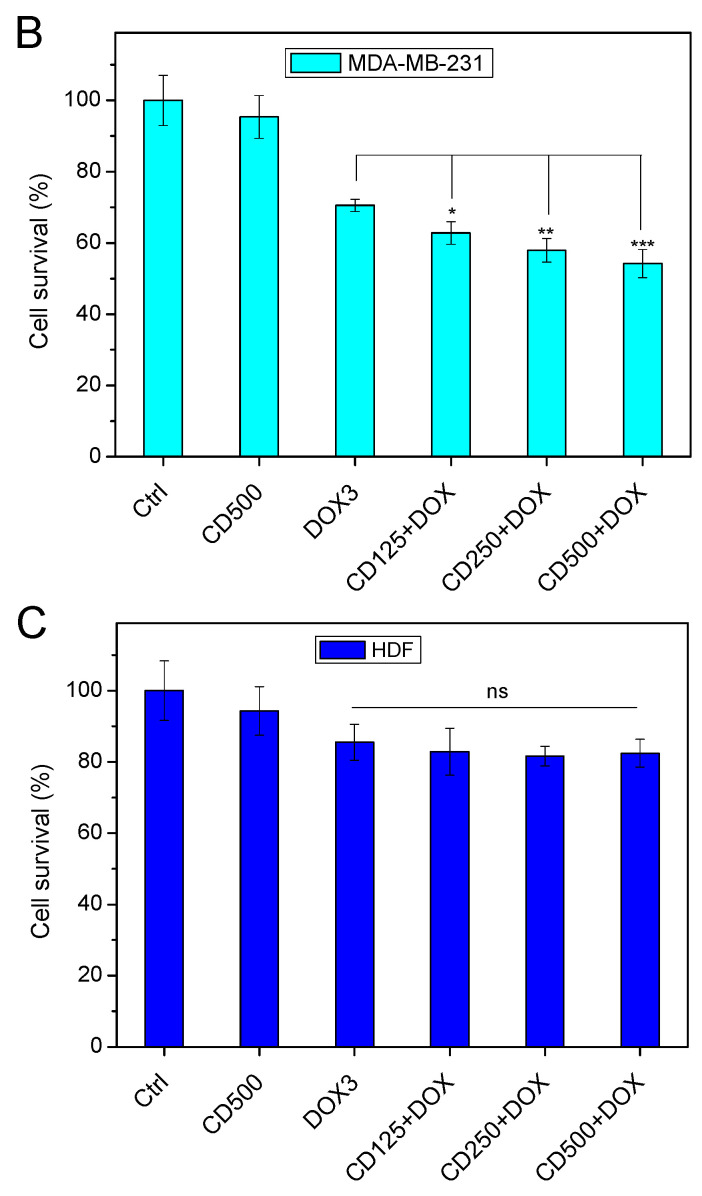
Carbon dots sensitizes p53 WT (MCF-7) and mutant p53 breast cancer (MDA-MB-231) cell lines but not human dermal fibroblasts (HDF) to DOX. Cells were treated with CDs (500 μg/mL), DOX (3 μΜ), as well as with increasing concentrations of CDs (125, 250, and 500 μg/mL) for 1 h before adding DOX (3 μM). After 24 h, cell survival of MCF-7 (**A**), MDA-MB-231 (**B**) and HDF (**C**) cell lines was measured with MTS assay. Results are expressed as the mean ± SD for at least three independent experiments and analyzed using Student’s *t*-test (* *p* < 0.05, ** *p* < 0.01, *** *p* < 0.001, ns not significant).

To examine whether the observed DOX-internalization increase had a significant impact on cell viability, we comparatively investigated the cell survival of DOX on MCF-7 cells and the non-cancerous human dermal fibroblasts (HDF). Furthermore, given that the p53 status has an important role in regulating DOX sensitivity of breast cancer resistant cells [63,64,65], we examined whether a breast cancer cell line expressing mutant p53 yields a different response. To this end, we also compared DOX cytotoxicity against the cell line MCF-7, which expresses the wild-type p53, to MDA-MB-231 cells that express the tumor suppressor gene p53 mutant R280K. As shown in Figure 6A,B, the viability of both breast cancer cell lines was equally affected by the presence of CDs in a concentration-dependent way. Viability in the presence of free DOX was, in both cases, close to 70% and gradually decreased in the presence of CDs to ~50%. On the other hand, DOX had a minor effect on the non-cancerous cells (Figure 6C), as in that case, the presence of CDs only affected cell viability to a very small extent, which was non-statistically significant, in line with the registered cell-internalization results obtained with this cell line.

## 3. Discussion

To rationalize the observed changes in the liposomal membrane characteristics in the presence of CDs and to gain insight into the interaction of CDs with the bilayer, we tentatively resorted to the well-studied effects of proteins on the properties of phospholipid bilayers. This was justified due to the current lack of a complete survey of nanoparticles–phospholipid membrane interactions, in contrast to the corresponding protein–membrane interactions, as well as due to the distant resemblance of the nitrogen-doped CDs employed in this study with proteins, in the sense that they have an almost spherical shape and are also characterized by the presence of amino acid, carboxylic acid, and hydroxyl groups on their surface. Specific lipid–protein interactions are important in understanding biological systems, especially regarding the activity of integral and peripheral membrane proteins and, thus, extensive studies were conducted on the complex nature of the interactions between proteins and lipids [66]. Research was primarily focused on the role of phospholipid membranes on the transport properties of cell membranes and how proteins might interact and affect lipid fluidity. A generally useful classification system was proposed by Papahadjopoulos [67], in which all proteins fall into one of three groups, according to their characteristic effects on phospholipids phase transitions and permeability. Although this classification scheme is not fully adequate to describe the effects of all naturally occurring membrane proteins [58], our results for the CD–lipid system studied in this work fall well within one of the three groups of interactions. Indeed, the observed large increase in the permeability of the liposomal bilayer, the minor change in *T*_m_ or in the width of the main phase transition, and the decrease in the enthalpy of the transition with increasing protein concentration suggest that the CD–lipid interaction follows the so-called Type 3 interaction. 

According to this classification, the nanoparticles are localized partly within the bilayer, where the hydrophobic part of the nanoparticle is situated in the lipophilic region of the bilayer, while their polar groups are located close to the polar surface of the lipid bilayer [58,67]. In a similar, almost parallel, approach, analysis of the heat capacity profiles by Monte Carlo simulations exploring lipid–protein interactions proposed that when the *c*_p_ maximum does not shift either to higher or lower temperatures, the peptides do not mix with both lipid phases, suggesting their accumulation at the gel–fluid domain interfaces [68]. It is, therefore, reasonable to propose that the nanoparticles are preferably localized in the boundaries between highly ordered phospholipid domains of the membrane [38,69,70], as there is no significant change in the transition temperature or in the width of transition even at high nanoparticle concentrations. It should also be added that this proposed model is compatible with the sizes of CDs that are found to be about 4.5 nm, smaller than the reported thickness (d = 69.7 Å) of a hydrated dipalmitoyl phosphatidylcholine lamella [71].

In vitro cell culture experiments suggest that in the presence of CDs, DOX is preferably internalized and is more cytotoxic against cancerous than against non-cancerous cells. The presence of CDs in the medium results in sensitization to DOX treatment in a dose-dependent fashion for both MCF-7 and MDA-MB-231 human breast cancer cell lines, which is not the case for the HDF human dermal fibroblasts. The observed difference can be attributed to the difference in cancer cells’ surface charge. It is well established that cell membrane charge increases during tumorigenesis because cancer transformation also alters the lipid bilayer membrane [60,61]; especially for human breast cancer cells, an increased amount of phospholipids was observed, which can increase the surface density of negatively charged groups [62]. In this context, it has been shown that while in eukaryotic cells the negatively charged phospholipids, mainly phosphatidylserine, are primarily located in the inner part of the bilayer leaflet, in cancerous cells their presence in the outer part of the membrane is increased [72,73,74]. In addition, the surface charge of cancer cells is also ascribed to the presence of the negatively charged, glycosaminoglycans, whose synthesis is increased during tumorigenesis [39], and also to the increased content of sialic acid in their glycolipids and glycoproteins [62,75]. Alternatively, the elevated negative charge of cancerous cells’ membranes has been attributed to the elevated glycolysis in the cancer cells that leads to increased secretion of lactate ions and, ultimately, to the high concentration of the negative surface charge [76]. Although it is not yet clear which of the above biochemical pathways primarily contributes to the elevated negative cell surface charge, this unique property of cancer cells is used to justify the cancer cell targeting ability, the cellular internalization, and the elevated cytotoxicity of various positively charged nanoparticles, as well as to rationalize the label-free separation of circulating tumor cells from blood [76,77,78,79,80,81,82]. Our cell culture results also point to the electrostatic interaction of positively charged CDs with the negatively charge membrane of cancer cells that, as the physicochemical studies with phospholipid bilayers suggest, could explain the observed increase in the cell membrane permeabilization and the concomitant increased DOX internalization and cytotoxicity.

## 4. Materials and Methods

### 4.1. Materials

Citric acid (99.8%) and ethylenediamine (≥99%) were purchased from Sigma–Aldrich Ltd. (Poole, UK). The phospholipids 1,2-dipalmitoyl-*sn*-glycero-3-phosphocholine (DPPC) and 1,2-dipalmitoyl-sn-glycero-3-phospho-rac-glycerol sodium salt (DPPG) were obtained from Lipoid GmbH (Ludwigshafen, Germany), while 1,2-distearoyl-*sn*-glycero-3-phosphoethanolamine-N-[methoxy(polyethylene glycol)-2000] ammonium salt (DSPE-PEG) was obtained from Avanti Polar Lipids (Alabaster, AL, USA). Doxorubicin hydrochloride (DOX) was kindly donated by Regulon SA (Athens, Greece). Nucleopore filters of 100 nm pore size (Whatman, Maidstone, UK) were employed for liposome extrusion. Sephadex G-50 (medium) and thiazolyl blue tetrazolium bromide (MTT) were obtained from Sigma–Aldrich (St. Louis, MA, USA). Cell culture RPMI 1640 medium with L-glutamine, fetal bovine serum (FBS), penicillin/streptomycin, phosphate buffer saline (PBS), and trypsin (0.05% *w*/*v*) /EDTA (0.25% *w*/*v*) were purchased from Invitrogen Ltd. (Paisley, UK). All other reagents and solvents were of analytical grade and used without further purification.

### 4.2. Synthesis and Characterization of N-Doped CDs

The preparation and detailed characterization of N-doped CDs from citric acid and ethylenediamine was described extensively in our previous publication [18]. In short, citric acid and ethylenediamine, at a molar ratio of 0.90:1, were dissolved in water and heated in a microwave oven (800 W) for 2 min. The resulting products were dissolved in water, filtered through a 0.22 μm syringe filter, extensively dialyzed against doubly distilled water employing a dialysis membrane of MW 12,000 g mol^−1^ cut-off, and lyophilized. The resulting CD nanoparticles with carboxylic, hydroxyl, and amino groups located at their surface had a size of about 4.5 nm (see the TEM images in Figure S1) and a z-potential of 2.5 ± 1.2 mV at pH 7.4 (phosphate buffer saline), while their maximum fluorescence was observed at 460 nm (λ_ex_ = 362), with a quantum yield of 49% [18].

### 4.3. Liposomes Preparation

Small unilamellar DPPC:DPPG:DPSE-PEG liposomes were prepared by the extrusion method (Olson et al., 1979) employing a LiposoFast-Pneumatic laboratory extruder (Avestin Inc., Ottawa, ON, Canada) [83]. In a typical experiment, for the preparation of 2.4 mL liposomal dispersion, 23.5 mg of DPPC, 1.2 mg of DPPG (5% molar with respect to DPPC), and 4.5 mg of DSPE-PEG (5% molar with respect to DPPC) were dissolved in a chloroform/methanol solution (2:1 *v*/*v*). The solvents were evaporated in a rotavapor (30–32 °C) for the formation of lipid film, which was then further held under high vacuum overnight to remove residual solvent traces and, subsequently, hydrated with 2.4 mL of a PBS solution (50 °C; 30 min). The obtained suspension was extruded through two stacked polycarbonate filters of 100 nm pore size. Twenty-five cycles were applied at 50 °C. For the accurate determination of the total lipid concentration in the final preparations, 500 μL of liposomal dispersions were ultracentrifuged (90,000 rpm, 45 min, 18 °C, Optima^TM^ Max Ultracentrifuge coupled with the MLA-130 rotor, Beckman Coulter, Inc., Fullerton, CA, USA) and the resulting pellet was lyophilized. Subsequently, the molar concentration of total lipids in each sample was determined by employing ^1^H NMR spectroscopy, using naphthalene as an internal standard (for details see the Supplementary Materials, Figure S2). The actual final total lipid concentration of the preparations was 11.5 ± 0.4 mg/mL.

### 4.4. Quantitative Analysis of CDs Associated with the Liposomes

CDs were initially dissolved in PBS solution to a final concentration of 1.5 mg/mL. Appropriate volumes were added to liposomal solutions to obtain liposomal dispersions with nominal CDs concentrations of 150, 250, and 500 μg/mL and allowed to incubate at room temperature for 1 h. For the determination of CDs associated with the liposomal membrane, the liposomal dispersions were ultracentrifuged, as described above, to remove non-interacted CDs. The resulting pellets were washed with PBS to ensure complete removal of CDs and, finally, completely dissolved in t-butanol:PBS (1:1). The concentration of CDs in the final solutions was determined by fluorescence spectroscopy, registering the intensity at 446 nm (λ_ex_ = 363 nm) and employing a separately constructed calibration curve of CDs (1–10 μg/mL) in t-butanol:PBS (1:1).

### 4.5. Measurement of the Permeability of Bilayer Membranes

DOX release from liposomal membranes was used to study the effect of CDs on negatively charged lipid bilayers. The release of DOX was manifested as an increase in the fluorescence intensity of DOX upon dilution in the outer bulk phase of self-quenched DOX encapsulated in DPPC:DPPG:DPSE-PEG liposomes.

#### 4.5.1. Liposomal DOX Encapsulation

DOX encapsulation was attained via the pH gradient active-loading method [84,85,86]. In short, the prepared DPPC:DPPG:DSPE-PEG dry lipid film was hydrated at 50 °C with 2.4 mL of isotonic (157 mM) citric acid buffer, pH 4.0, and extruded as described above. The concentration of the citric acid in the buffer was chosen so as to have the same total osmolality as that of PBS (300 mOsm/L). Following extrusion and cooling at room temperature, the external aqueous solution of the liposomal dispersions was exchanged with PBS buffer by passing the liposomes through a Sephadex G-50 size exclusion minicolumn (conditioned in PBS, pH 7.4). DOX (1 mg) was added in the liposomal dispersion and kept at 37.5 °C for 40 min with gentle stirring under an argon atmosphere. Finally, the liposomal dispersions were cooled to room temperature and passed through a Sephadex G-50 medium minicolumn (conditioned in PBS, pH 7.4) to remove non-encapsulated DOX. The encapsulated DOX concentration was determined by employing a Cary Eclipse fluorescence spectrophotometer (Mulgrave, VIC, Australia), registering the fluorescence intensity at 592 nm (λ_ex_ = 500 nm) of a known amount of liposomal dispersion in PBS after the addition of 25 μL of Triton X-100 (20% *w*/*w*) solution for the complete solubilization of liposomes and the release of encapsulated DOX. To this end, a separately constructed calibration curve of DOX (1–10 μM) in PBS was used. DOX concentrations were 0.60 ± 0.10 mM (0.35 mg/mL) and the drug/total lipid ratio was 2.9–3.0 wt.%). All liposomal samples were sterile filtered (0.22 μm, Millipore, Danvers, MA, USA) and either used immediately or stored at 4 °C and used within the next day.

#### 4.5.2. Time-Dependent Release of Encapsulated DOX

DOX release from liposomes was based on the self-quenching property of DOX at high concentrations (when it was located in the liposomal interior) while exhibiting strong concentration-dependent fluorescence at low concentrations (i.e., following its release from the liposomes to the medium). Thus, the released DOX concentration was monitored by continuously registering the fluorescence intensity of dispersions using the Cary Eclipse fluorescence spectrophotometer coupled with a Cary Single Cell Peltier accessory (type SPVF—1x0) that was able to continuously stir and stabilize the temperature in the cell with 0.1 °C precision. Typically, 30 μL of DOX-loaded liposomes were added in 2700 μL of PBS solution, which was thermally equilibrated in the fluorescence cell at the pre-determined temperature. After the addition of liposomes in the already heated buffer medium, the monitoring of the fluorescence intensity was immediately initiated, typically within 10 s from the addition. The fluorescence intensity over time, I_t_, of the released DOX was continuously monitored for 40 min (excitation wavelength = 500 nm, emission wavelength = 592 nm). After 30 min, 25 μL of 10% Triton X-100 was added in order to solubilize the liposomes and drive all DOX in the aqueous media to obtain I_max_ at each specific temperature. DOX release was calculated as Release (%) = (I_t_ − I_o_)/(I_max_ − I_o_) × 100. The initial fluorescence intensity, I_o_, was determined at 25 °C in a separate experiment conducted as above. As the release rate of drugs from liposomes is typically proposed to follow first order kinetics [87,88,89,90], the apparent rate constants, *k*, of DOX was calculated by fitting the obtained data points, using the first order kinetics equation I_t_/I_max_ = 1 − e^A−*k*t^, where A = ln(1 − I_o_/I_max_).

### 4.6. Characterization Techniques

The obtained DPPC:DPPG:DSPE-PEG liposomes before and after their interaction with CDs were investigated by dynamic light scattering, z-potential experiments, and temperature-modulated differential-scanning calorimetry (MDSC), while the permeability of the bilayer at various temperatures was assessed by monitoring the release of DOX (see Section 2.4). The mean hydrodynamic radii and size distribution of liposomal dispersions in the presence of increasing amounts of CDs were determined at 25 °C by dynamic light scattering employing an AXIOS-150/EX apparatus (Triton Hellas, Thessaloniki, Greece) equipped with a 30 mW laser source emitting at 658 nm and an Avalanche photodiode detector at an angle of 90°. For these experiments, 50 μL of liposomes were diluted with 0.2 mL of PBS buffer. Ten scattering measurements were acquired for each dispersion and the results were averaged. Autocorrelation functions were collected for 20 s and analyzed using the CONTIN algorithm to obtain the apparent hydrodynamic radii distribution.

^1^H NMR spectra were recorded in CDCl_3_ by a Bruker Avance DRX spectrometer operating at 500 MHz. The fluorescence spectra were recorded on a Cary Eclipse fluorescence spectrophotometer from Varian Inc. (Mulgrave, VIC, Australia). UV-Vis spectra were recorded using a Cary 100 Conc UV−visible spectrophotometer (Varian Inc.). Transmission electron microscopy (TEM) experiments were performed to investigate the size and morphology of the respective nanoparticles employing an FEI Talos F200i field-emission (scanning) transmission electron microscope (Thermo Fisher Scientific Inc., Waltham, MA, USA) operating at 200 kV, equipped with a windowless energy-dispersive spectroscopy microanalyzer (6T/100 Bruker, Hamburg, Germany). In this case, a droplet of water CDs solution was deposited on a carbon-coated 200 mesh copper grid and allowed to evaporate in air.

The z-potential values were obtained at 25 °C using ZetaPlus of the Brookhaven Instruments Corp. (Long Island, NY, USA), equipped with a 35 mW solid-state laser emitting at 660 nm. From the obtained electrophoretic mobility, the z-potential of the liposomal dispersions was calculated using Smoluchowski’s equation. In a typical experiment, 50 μL of a liposomal dispersion was diluted with 1.5 mL of PBS and introduced into the instrument cell. Ten measurements were collected for each dispersion and the results were averaged.

The main lipid phase transition was assessed by Temperature Modulated DSC (MDSC) for the DPPC:DPPG:DSPE-PEG liposomal formulations in the presence of increasing amounts of CDs in the outer aqueous phase. DSC measurements were performed by employing a MDSC 2920 calorimeter (TA Instruments, New Castle, DE, USA) under nitrogen flow (20 mL/min), using a heating/cooling rate of 2 °C/min, a temperature modulation amplitude of 0.310 °C every 60 s, and an empty pan as a reference. For reproducibility assessment, two heating/cooling scans were carried out from 15 °C to 60 °C and the transition temperature, *T*_m_, taken as the center of the main lipid transition peak, as well as the transition enthalpy, ΔH, were determined using the in-built software. Before each heating/cooling scan, the sample solution was equilibrated at the respective starting temperature for 5 min. In all cases, the error of at least 3 different batches was less than ±0.2 °C. Heat and temperature calibrations were performed by using indium as a standard. For each experiment in 300 μL of liposomal dispersions, appropriate volumes of CD solution (1.5 mg/mL) were added in the external outer phase to afford liposomal dispersions with different CDs concentrations (0, 125, 250, 500 μg/mL). The dispersions were ultracentrifuged (90,000 rpm, 45 min, 18 °C) in an Optima^TM^ Max Ultracentrifuge Beckman Coulter, Inc. (Fullerton, CA, USA) coupled with the MLA-130 rotor. The resulting wet pellet (typically 10 mg) was transferred, accurately weighted, hermetically sealed into aluminum pans, and transferred to the calorimeter [91]. Independently, 500 μL of liposomal dispersions of the above samples were ultracentrifuged as above and the resulting pellet was lyophilized and, subsequently, the molar concentration of total lipids in each sample was determined employing ^1^H NMR spectroscopy, as described in Section 2.3.

### 4.7. Cell Culture and Treatments

Cells used in this study were the human breast cancer cell lines MCF-7 and MDA-MB-231, as well as the non-cancerous human dermal fibroblasts (HDF), obtained from the cell bank of the Institute of Biosciences and Applications, NCSR “Demokritos”. The cells were grown in RPMI 1640 medium with stable glutamine, supplemented with 10% FBS and 1% penicillin/streptomycin at 37 °C in a 5% CO_2_ humidified atmosphere.

### 4.8. Cellular Uptake of DOX

Cells were seeded into 96-well plates at a density of 15 × 10^3^ cells per well and left to grow overnight. The cells were treated with increasing concentrations of CDs (125, 250, 500 μg/mL) for 1 h before adding DOX (3 μM). In addition to the control, we also had a number of wells with cells treated only with DOX at the same concentration. After 3 h, the wells were washed with RPMI (without phenol red) and DOX internalization was monitored by measuring the fluorescence intensity using a Tecan plate reader (Infinite M200 plate reader, Tecan, Männedof, Switzerland, λ_ex_ = 510 nm, λ_em_ = 580 nm). The employment of the colorless medium RPMI (absence of phenol red) was necessary in order to avoid any spectral interference with the final measurement of DOX.

### 4.9. Cytotoxicity Assay

Cells were seeded into 96-well plates at a density of 10 × 10^3^ cells per well. The following day, the cells were treated with different concentrations of CDs with or without DOX (1, 3 μM) at 37 °C in complete medium for 24 h, and the mitochondrial redox function of all cell groups was assessed by the MTT assay (Sigma, Roedermark, Germany). Briefly, the cells were incubated with 1 mg/mL MTT-containing medium for 4 h and, following MTT removal, the produced formazan crystals were solubilized in isopropanol. The absorbance was measured with an Infinite M200 plate reader (Tecan group Ltd., Männedorf, Switzerland) at a wavelength of 570 nm.

### 4.10. Statistical Analysis

All experiments were repeated independently at least three times. Cellular uptake and MTT data are shown as means of six independent values with error bars representing standard deviation. Student’s *t*-test was performed on the data obtained to determine the statistical significance of a difference between means. Τhe statistical significance followed the assignment: * *p* < 0.05, ** *p* < 0.01, *** *p* < 0.001, and **** *p* < 0.0001.

## 5. Conclusions

To obtain insight on the effect of nitrogen-doped carbon dots (CDs) on living cells, we studied their effect on the structural, thermodynamic, and permeability properties of a model liposomal system. It was found that CDs interact electrostatically with the model membrane, affecting the size, the z-potential, and the thermodynamics of lipid bilayer melting. Together with the registered substantial increase in membrane permeability, as manifested by the fast and concentration-dependent release of liposomal encapsulated DOX, the results suggested that CDs are positioned at least partly within the phospholipid bilayer, most probably between the crystalline microdomains. Even though the complexity of biological systems is such that one should expect that a wide variety of factors will influence nanoparticle interactions with cell membranes, our initial in vitro experiments with cancerous and non-cancerous cell lines corroborated the primarily electrostatic nature of CDs’ interactions with cancerous cells and the observed increase in membrane permeability, as enhanced DOX internalization -and concurrent cytotoxicity was observed. While further in vitro studies are needed, it is important to highlight the possible effect on cell membranes of the otherwise benign carbon dots and the indirect effect their presence might have on living systems. In addition, CDs can be favorably employed as DOX-sensitizers, and ways to harness their usefulness in this respect must be pursued.

## Figures and Tables

**Table 1:** Apparent release rate constants, *k*, of DOX from DPPC:DPPG:DSPE-PEG liposomes calculated from the temperature-dependent release profile presented in Figure 4, the diffusional release exponent, n, derived from the Korsmeyer–Peppas equation, and total DOX release within the first 40 min.

CD Concentration (μg/mL)	*k* (min^−1^) ^a^	Diffusional Release Exponent, n ^b^	Total DOX Release (%)
0	23.4 ± 1.5	0.47 ± 0.3	18.5 ± 0.5
125	26.5 ± 0.5	0.42 ± 0.3	27.5 ± 0.6
250	36.3 ± 1.0	0.47 ± 0.3	33.6 ± 0.6
500	54.9 ± 1.0	0.59 ± 0.5	40.8 ± 1.0

^a^ R-square > 0.998; ^b^ for the calculation of n, the last 30 min of release profiles were used.

## Data Availability

Data is contained within the article the Supplementary Materials.

## Supplementary Materials

The following supporting information can be downloaded at: https://www.mdpi.com/article/10.3390/ph16060833/s1, Figure S1: TEM images of N-doped CDs; Figure S2: 1H NMR spectrum of the lipids of DPPC:DPPG:DPSE-PEG liposomal dispersions; Figure S3. Heat capacity profiles obtained during the first heating cycle of liposomal DPPC:DPPG: DSPE-PEG formulations in the presence of various CDs concentrations; Figure S4: Drug release plotted vs. the square root of time and fitting of DOX release data using the Korsmeyer–Peppas equation.
