# Supplementary material for: Carbon Dots–Biomembrane Interactions and Their Implications for Cellular Drug Delivery
Source: Pharmaceuticals (Basel). 2023 Jun 2;16(6):833. doi: 10.3390/ph16060833 (PMC10305404; doi:10.3390/ph16060833)
Supplement: Supplementary file 1 [file pharmaceuticals-16-00833-s001.zip › pharmaceuticals-2413661-supplementary.pdf]

Carbon dots-biomembrane interactions and their implications on cellular drug delivery

Barbara Mavroidi [1], Archontia Kaminari [2], Elias Sakellis [2], Zili Sideratou [2] and Dimitris Tsiourvas [2,*]

[1] National Centre for Scientific Research “Demokritos”, Institute of Biosciences and Applications, 15310 Aghia Paraskevi, Greece; alexandr@bio.demokritos.gr
[2] National Centre for Scientific Research “Demokritos”, Institute of Nanoscience and Nanotechnology, 15310 Aghia Paraskevi, Greece; a.kaminari@inn.demokritos.gr (A.K.); e.sakellis@inn.demokritos.gr (E.S.); z.sideratou@inn.demokritos.gr (Z.S.)
*Correspondence: d.tsiourvas@inn.demokritos.gr; Tel.: +30-210-650-3616 (D.T.)

Supporting material

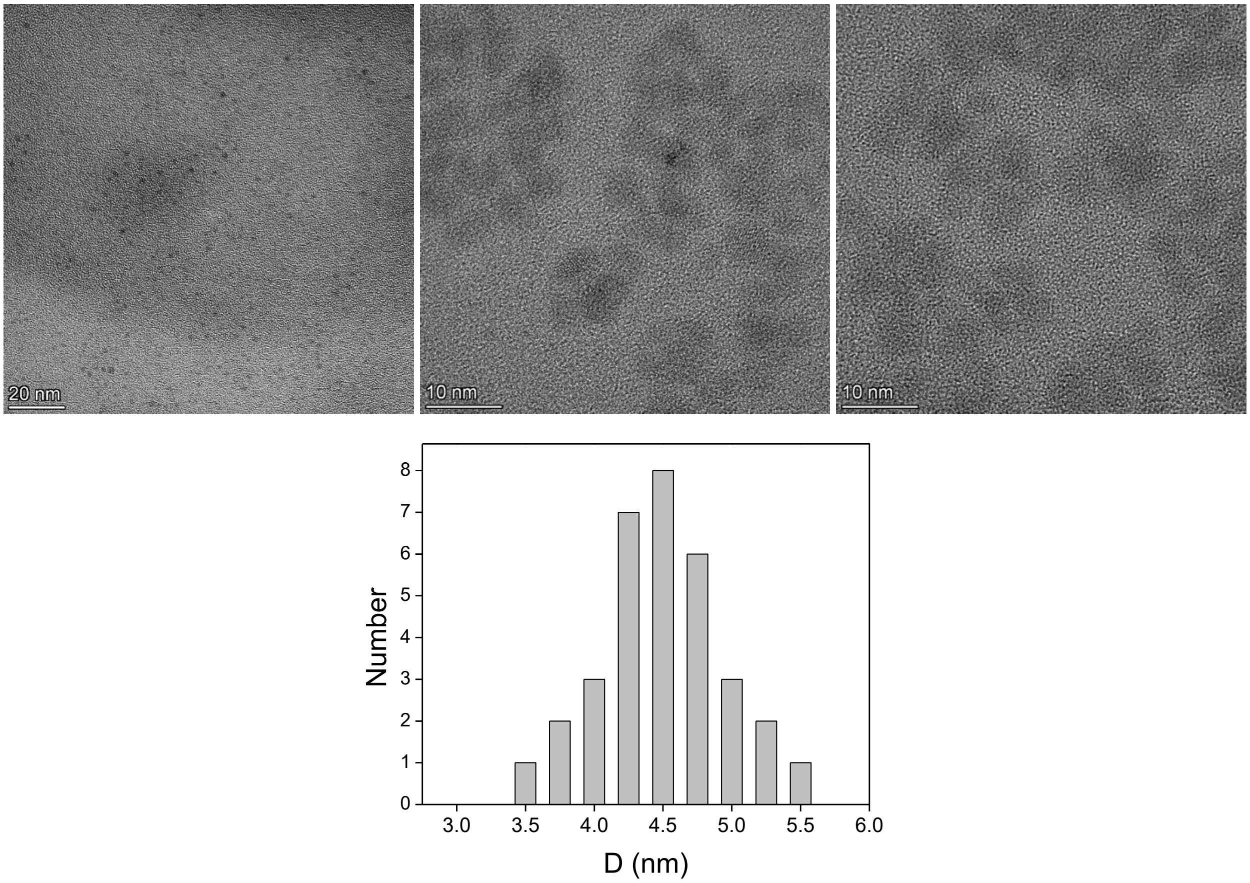

Figure S1. TEM images of N-doped CDs. Upper part: Typical images of the nanoparticles at two different magnifications. Lower part: number histogram showing particle size distribution.

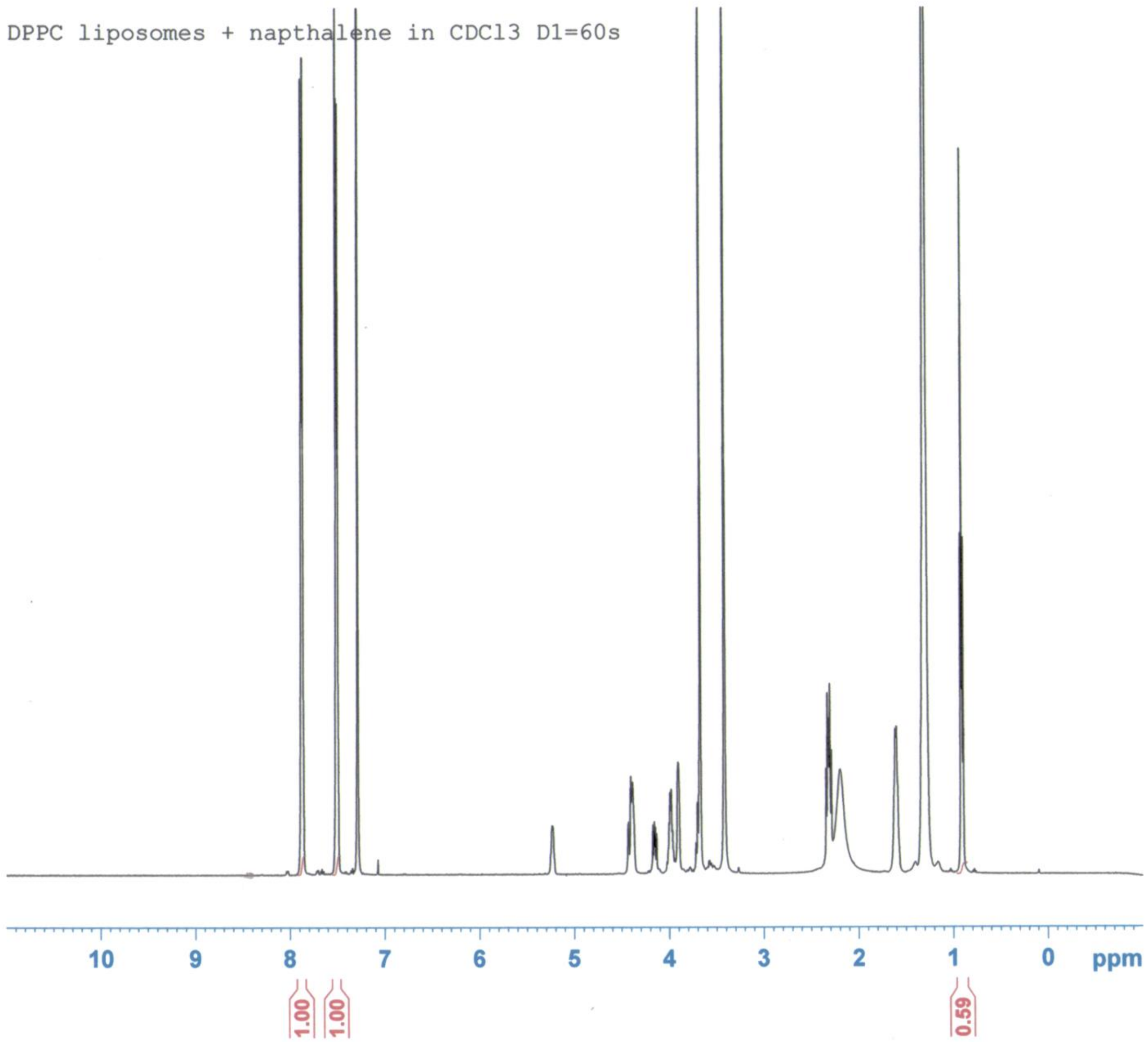

Figure S2. ^{1}H NMR spectrum of the lipids of DPPC:DPPG:DPSE-PEG liposomal dispersions (500 μL) obtained after ultracentrifugation and lyophilization, together with a known amount of naphthalene (internal standard) dissolved in $CDCl_3$ (25 ^{o}C, Bruker Avance DRX spectrometer operating at 500 MHz: preacquisition delay of 7.5μs, 90° pulse length of 8 μs, relaxation delay of 60 s, 32 k data points, corresponding to an acquisition time of 4.09 s at a sweep width of 8012.8 Hz). Fourier transformation was performed after zero filling the data to 64 k time domain points and line broadening of 0.3 Hz. The actual final total molar lipid concentration of the prepared liposomes was determined by comparing the integrals of the peaks at (i) 0.85 ppm attributed to the protons of methyl groups of the lipids and (ii) at 7.85 and 7.50 ppm assigned to the inner α-protons and outer β-protons of naphthalene, respectively.

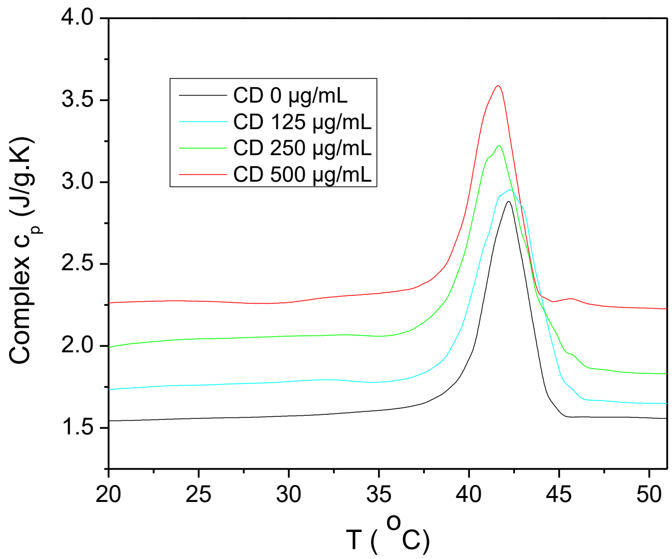

Figure S3. Complex heat capacity profiles obtained during the first heating cycle of liposomal DPPC:DPPG:DSPE-PEG formulations in the presence of various CDs concentrations in the outer PBS medium.

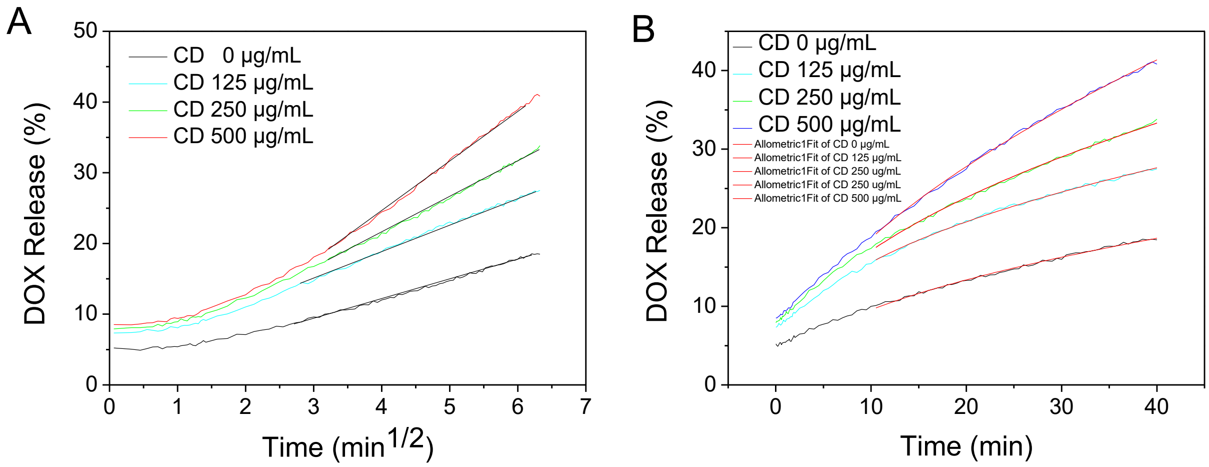

Figure S4. A: Drug release plotted vs. the square root of Time (Higuchi's law); linear relationship of release data is observed after ca. 10 min. B: Fitting of the DOX release data using the Korsmeyer-Peppas equation in the 10-40 min time range (adjusted R-square>0.996).
